# Proteomic Characterisation of the Salt Gland-Enriched Tissues of the Mangrove Tree Species *Avicennia officinalis*

**DOI:** 10.1371/journal.pone.0133386

**Published:** 2015-07-20

**Authors:** Wee-Kee Tan, Teck-Kwang Lim, Chiang-Shiong Loh, Prakash Kumar, Qingsong Lin

**Affiliations:** 1 Department of Biological Sciences, National University of Singapore, 14 Science Drive 4, Singapore, Singapore, 117543; 2 NUS Environmental Research Institute, National University of Singapore, 5A Engineering Drive 1, T-Lab, #02–01, Singapore, Singapore, 117411; Henan Agricultural Univerisity, CHINA

## Abstract

Plant salt glands are nature’s desalination devices that harbour potentially useful information pertaining to salt and water transport during secretion. As part of the program toward deciphering secretion mechanisms in salt glands, we used shotgun proteomics to compare the protein profiles of salt gland-enriched (isolated epidermal peels) and salt gland-deprived (mesophyll) tissues of the mangrove species *Avicennia officinalis*. The purpose of the work is to identify proteins that are present in the salt gland-enriched tissues. An average of 2189 and 977 proteins were identified from the epidermal peel and mesophyll tissues, respectively. Among these, 2188 proteins were identified in salt gland-enriched tissues and a total of 1032 selected proteins were categorized by Gene Ontology (GO) analysis. This paper reports for the first time the proteomic analysis of salt gland-enriched tissues of a mangrove tree species. Candidate proteins that may play a role in the desalination process of the mangrove salt glands and their potential localization were identified. Information obtained from this study paves the way for future proteomic research aiming at elucidating the molecular mechanism underlying secretion in plant salt glands. The data have been deposited to the ProteomeXchange with identifier PXD000771.

## Introduction

Plants growing in saline environment have to cope with the constant exposure to high levels of salt and limited availability of freshwater. In some halophytic plant species (i.e., plants that are able to tolerate salt concentrations as high as 500–1000mM), there exists specialized microscopic structures located predominantly on the leaves and stems that are able to remove salts from the internal tissues and deposit them on the leaf surfaces [1,2]. Known as the salt glands, they are nature’s desalination devices offering alternative routes for excess ion elimination through secretion, an adaptive feature that favours species inhabiting saline environment. Many of the salt gland studies focused on their secretory nature (e.g., [3–8]). The mechanism underlying such a desalination process, however, remains unclear.

Previous studies by us [9,10] focused on the salt glands of a commonly found mangrove tree species in Singapore (*Avicennia officinalis* L) [11,12]. By making use of an epidermal system developed, we discovered a unique secretory pattern [10] that had been considered as an example of high resolution measurements of secretions that may lead to a general understanding on the mechanism of fluid secretion in both plant and animal systems [13]. This species grows in intertidal zones and has to cope with periodic exposure to fluctuating salinities [14]. We hypothesize that salt glands function as salt and water bi-regulatory units and the salt glands of this species offer an excellent platform to investigate their dynamic responses and molecular underpinnings to fluctuating salinities. Modern high throughput proteomics tools allow more detailed quantitative information, both temporal and spatial expression of proteins, to be obtained [15]. Recent studies in search of salt-responsive proteins in mangroves have also adopted a proteomic approach [16–18]. These studies, however, focused on the non-secretors (i.e., *Bruguiera gymnorhiza*, *Kandelia candel*) that do not have salt glands on their epidermal surfaces. Due to technical challenges faced in obtaining proteins directly from salt glands, a recent proteomic paper published by our group [19] focused on the plasma membrane and tonoplast proteins extracted from the leaves of *A*. *officinalis*. All these studies reported thus far have adopted a gel-based analysis approach.

In this study, as a continuous effort to better understand how water and salt are transported via the salt glands, we have adopted a shotgun approach to look into the proteome of salt-gland enriched tissues of the mangrove tree species *A*. *officinalis*. By removing the bulk of the leaf tissues (i.e., mesophyll tissues) devoid of salt glands, proteins from tissues that are rich in salt glands could be successfully obtained. To compensate for the technical limitations in obtaining large amounts of proteins from salt-gland-rich tissues, the shotgun approach adopted in this study allows simplified handling of samples with more exhaustive digestion and avoidance of sample loss in the gel matrix [20]. The data obtained via this approach offers a glimpse into the proteome of salt gland-rich materials and serves as a platform for identifying pool of proteins that could be involved in the desalination process of the mangrove salt glands.

## Materials and Methods

### Plant materials and protein extraction

The leaves of *A*. *officinalis* were required for the isolation of salt gland-enriched tissues (i.e., adaxial epidermal peels) for subsequent protein extractions. Shoots of *A*. *officinalis* were first collected from the mangrove swamp at Berlayer Creek (Sungei Berlayer, Labrador, Singapore; 1.27°N; 103.80°E; permit for collection granted by Keppel Club, Singapore). For each biological replicate, the adaxial epidermal peels, which harbour the salt glands, were separated from the mesophyll tissues of ~20 leaves collected from several shoots according to Tan *et al*. [9]. Briefly, abaxial epidermal layers of excised leaves were removed, the leaves cut into segments before the leaf strips floated on enzyme mixture (pH 5.7; filter-sterilized) containing 0.1% (w/v) Pectolyase Y-23 (Seishin Pharmaceutical, Japan), 1.0% (w/v) Driselase (Sigma-Aldrich, USA) and 1.0% (w/v) Cellulase Y-C (Kikkoman Corporation, Japan) were vacuum infiltrated for 10 min and incubated in the dark at 30°C, 30 rpm for 1 h. The adaxial epidermal peels were easily detached from the mesophyll tissues after enzyme treatment. These peels were then rinsed, the remnants of mesophyll-palisade layers gently scraped off using a scalpel to obtain adaxial peels devoid of chlorophyll-containing cells and were collected separately from the mesophyll tissues. Three biological replicates were prepared. Total protein was extracted separately from these tissues by grinding them in liquid nitrogen and resuspending in buffer containing 25mM triethylammonium bicarbonate, 8M urea, 2% Triton X-100 and 0.1% sodium dodecyl sulphate [21]. The samples were then sonicated on ice for 30min, centrifuged (16000×g) at 15°C for 1h before supernatants were collected. Proteins were estimated using RCDC Protein Assay Kit (BioRad, Hercules, CA, USA) to compensate for interfering compounds in the samples.

### Sample preparation and LC-MS/MS analysis

Each sample (300μg) was reduced by 5mM tris(2-carboxyethyl)phosphine (Sigma-Aldrich, St. Louis, MO, USA) at room temperature for 1h and alkylated with 10mM methyl methanethiosulfonate (Sigma-Aldrich, St. Louis, MO, USA) at room temperature for 10min. The samples were then trypsin-digested (Promega, Madison, WI, USA) overnight at 37°C in a trypsin-to-protein ratio of 1:20 (W:W).

The first dimension peptide separation, which included removal of SDS and other contaminants, was carried out on a LC-10A1 Prominence Modular HPLC (Shimadzu Corporation, Japan). The digested sample (100μg) was diluted with 5ml strong cation-exchange mobile phase A [10mM potassium phosphate in 25% acetonitrile (ACN), pH 3.0] before solution was passed through a 3μm PolySULFOETHYL A column (35mm × 4.6mm; PolyLC Inc., Columbia, MD). Peptides were separated by gradient formed by mobile phase A and B (500mM KCl and 10mM potassium phosphate in 25% ACN, pH 3.0),: 0–0% mobile phase A in 10min, 0–36% mobile phase B in 80min, 36–70% mobile phase B in 30min, 70–100% mobile phase B in 1min, 100–100% mobile phase B in 10min and 0–0% mobile phase B in 10min, each at a flow rate of 0.5 ml/min. The digested peptides (100μg) separated were combined to 8 fractions (~12.5μg proteins/fraction), desalted with Sep-Pak Classic C18 cartridge (Waters, Milford, MA, USA), lyophilized before a second-dimension reversed-phase (RP) chromatography was carried out on Eksigent nanoLC Ultra and ChiPLC-nanoflex (Eksigent, Dublin, CA, USA).

Desalted samples were reconstituted with 15μl diluent [2% ACN, 0.05% formic acid (FA)], 5μl of which was loaded on 200μm × 0.5mm trap column and eluted onto analytical 75μm × 150mm column. Both columns were made of Repro-Sil-Pur C18-AQ, 3μm (Eksigent, Dr. Maisch, Germany). Peptides were separated by gradient formed by mobile phase A (2% ACN, 0.1% FA) and B (98% ACN, 0.1% FA): 5–12% mobile phase B in 20min, 12–30% mobile phase B in 40min, 30–90% mobile phase B in 2min, each at a flow rate of 300nl/min. The MS analysis was performed on TripleTOF 5600 system (AB SCIEX, Foster City, CA, USA) in Information Dependent Mode. MS spectra were acquired across mass range of 400–1800m/z in high resolution mode (> 30000) using 250ms accumulation time/spectrum. A maximum of 20 precursors/cycle was chosen for fragmentation from each MS spectrum with 100ms minimum accumulation time for each precursor and dynamic exclusion for 15s. Tandem mass spectra were recorded in high sensitivity mode (resolution > 15000) with rolling collision energy on.

Peptide identification and quantification was performed with ProteinPilot 4.5 software Revision 1656 (AB SCIEX) using the Paragon database search algorithm (4.5.0.0) and integrated false discovery rate (FDR) analysis function. The obtained MS/MS spectra were then searched against a database created [i.e., derived from transcriptome sequencing of salt gland–enriched tissues (i.e., adaxial epidermal peels of *A*. *officinalis*, with a total 174552 entries including both normal and decoy sequences)]. The following search parameters were adopted: Sample Type—Identification; Cys Alkylation—MMTS; Digestion—trypsin; Special Factors—None; Species—None. The processing was specified as follows: ID Focus—Biological Modifications; Search Effort—Thorough; Detected Protein Threshold—0.05 (10.0%). Identified proteins for each biological replicate were selected based on a false discovery rate (FDR) of < 1%.

For Gene Ontology (GO) studies [22], proteins identified in the salt gland-enriched tissues and that are present in at least two of the biological replicates were selected for further analysis. These selected proteins were first submitted to the UniProt Knowledgebase (UniProtKB) website (http://www.uniprot.org/help/uniprotkb) to retrieve the corresponding UniProtKB/Swiss-Prot entries and only annotated entities (i.e., with matched Swiss-Prot ID) were consolidated for GO analysis.

## Results

**Fig 1:**
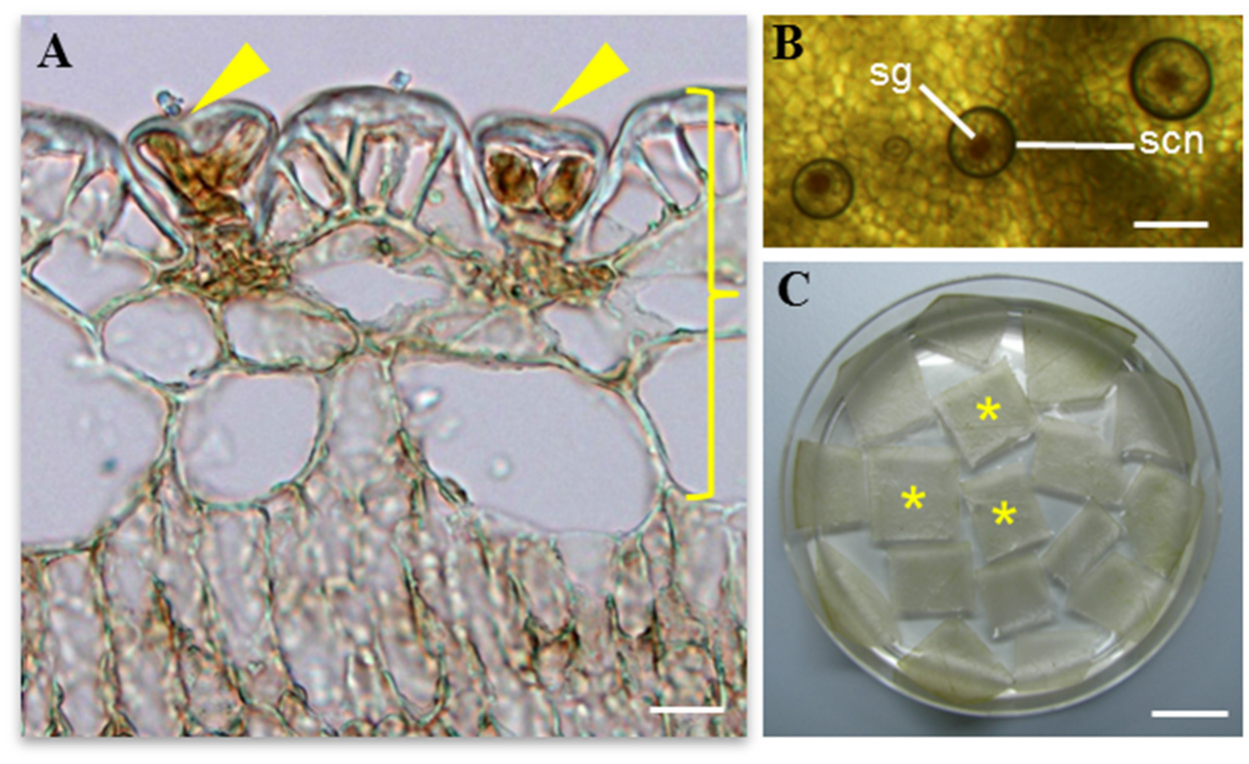
Salt glands of the mangrove species *Avicennia officinalis*. (A) Transverse section of leaf showing the adaxial (upper) epidermal layer with two salt glands (arrows). (B) Secretion (scn) above the salt gland (sg) can be observed from the top view of the adaxial epidermal layer. (C) The salt gland-enriched epidermal peels (*) as indicated by the right brace in (A) were obtained from the leaves for subsequent protein extraction and downstream proteomic analysis. Scale bars: 20μm (A), 100μm (B), 1cm (C).

**Fig 2:**
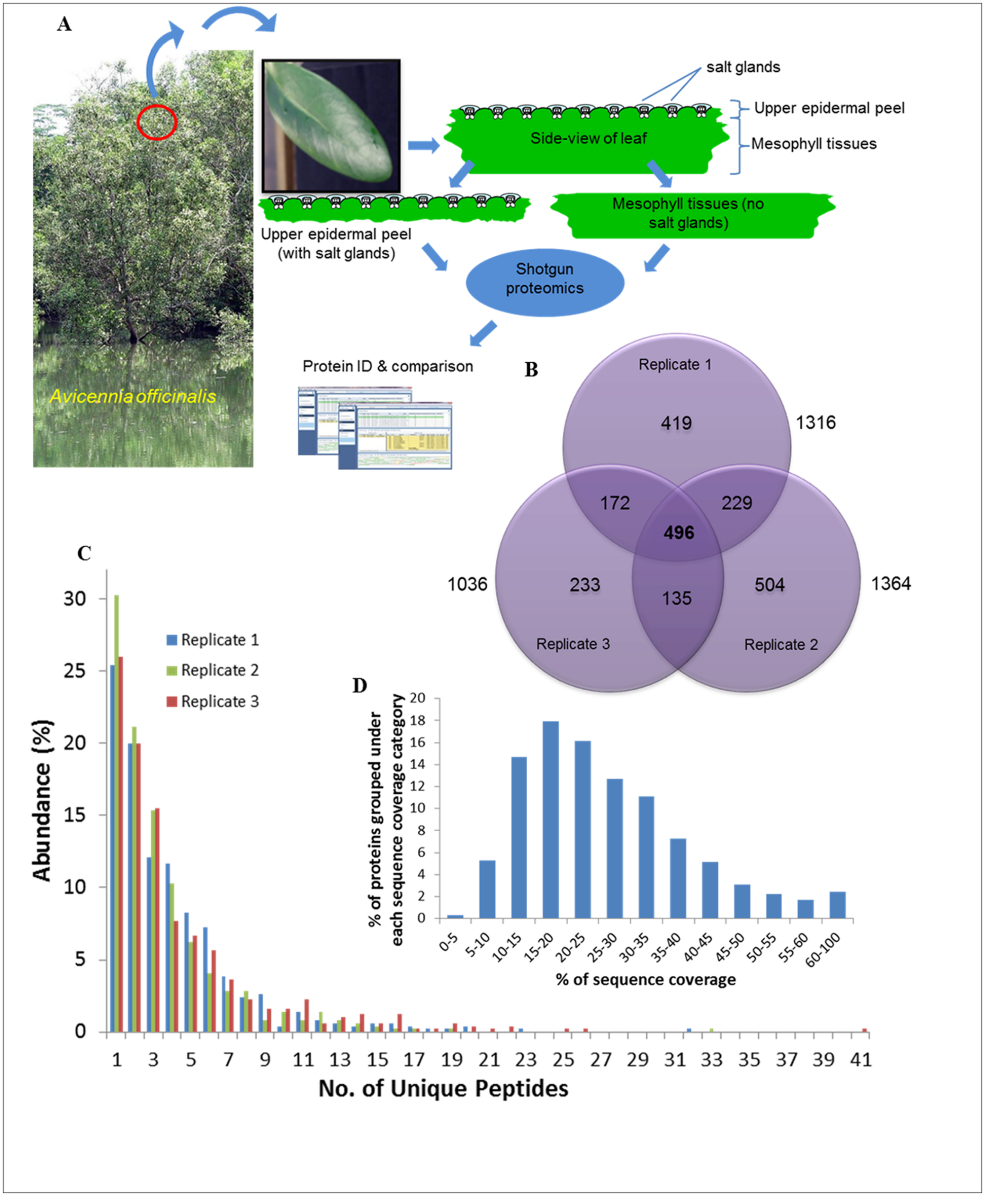
Identification and analysis of salt gland-enriched proteome. (A) The experimental approach for generation of a salt gland-enriched proteome through the use of two distinct set of samples: total proteins from the adaxial (upper) epidermal peels (with salt glands) and from the mesophyll tissues (no salt glands). (B) The number of proteins that are identified in salt gland-enriched epidermal peels from three biological replicates is presented in the Venn diagram. Identified proteins from the salt gland-enriched tissues that were present in all the three biological replicates were grouped according to the number of unique peptides (C) and % sequence coverage (D). The identified proteins (D) were classified according to the protein’s sequence coverage.

The salt glands of *Avicennia officinalis* are microscopic (20–40 μm) structures found on the epidermal leaf surfaces (Fig 1A). They can secrete droplets of salt solutions, which appear circular in shape above the salt glands under a layer of oil when the adaxial (upper) epidermal peel (which harbours the salt glands) was viewed from the top (Fig 1B). These adaxial epidermal peels that are enriched with salt glands (Fig 1C) thus serve as good starting materials for the study of the salt gland proteome. To achieve this, proteins from both the adaxial epidermal peels (salt gland-enriched) and mesophyll tissues (salt gland-deprived) were extracted and compared (Fig 2A). For each extraction, approximately 2 mg proteins/g tissues and 9 mg proteins/g tissues were obtained from the epidermal peels and mesophyll tissues, respectively. A 2DLC/MS/MS analysis was performed on each of the trypsin-digested samples and identified proteins for each biological replicate were selected (< 1% FDR; S1–S6 Tables). An average of 2189 ± 128 (Table 1 and S1–S3 Tables) and 977 ± 150 (Table 1 and S4–S6 Tables) proteins were observed from the epidermal peels and mesophyll tissues, respectively. To obtain a list of proteins from salt gland-enriched tissues, only proteins that are found in epidermal peels but not in mesophyll tissues were considered. Data were sorted using nwCompare [23], with proteins found in any biological replicates for each tissue type taken into consideration and those extracted from epidermal peels and can be identified from mesophyll tissues eliminated. Using this approach, 2188 proteins are identified in salt gland-enriched tissues (Fig 2B and see S7 Table). Of these, 496 proteins were commonly found in all biological replicates, 536 proteins observed in two out of three biological replicates while remaining 1156 were present in one of the biological replicates (Fig 2B). Among the 496 proteins that were commonly found in all three biological replicates analysed, more than 25% of the proteins with at least one unique peptide was identified, while ~50% of the proteins with 2–5 unique peptides and the rest with at least 6 unique peptides identified (Fig 2C). By looking at the distribution of protein sequence coverage, more than 65% of the proteins identified showed a sequence coverage of 15–40% (Fig 2D).

**Table 1:** Number of proteins identified from adaxial (upper) epidermal peels and mesophyll tissues of the leaves of *A*. *officinalis*.

	Average no. of proteins identified (±SE)
**Epidermal peels**	2189 ± 128
**Mesophyll tissues**	977 ± 150

Three biological replicates from each type of tissues were prepared and the protein profiles compared using a shotgun approach. Results are presented as mean ± SE.

**Fig 3:**
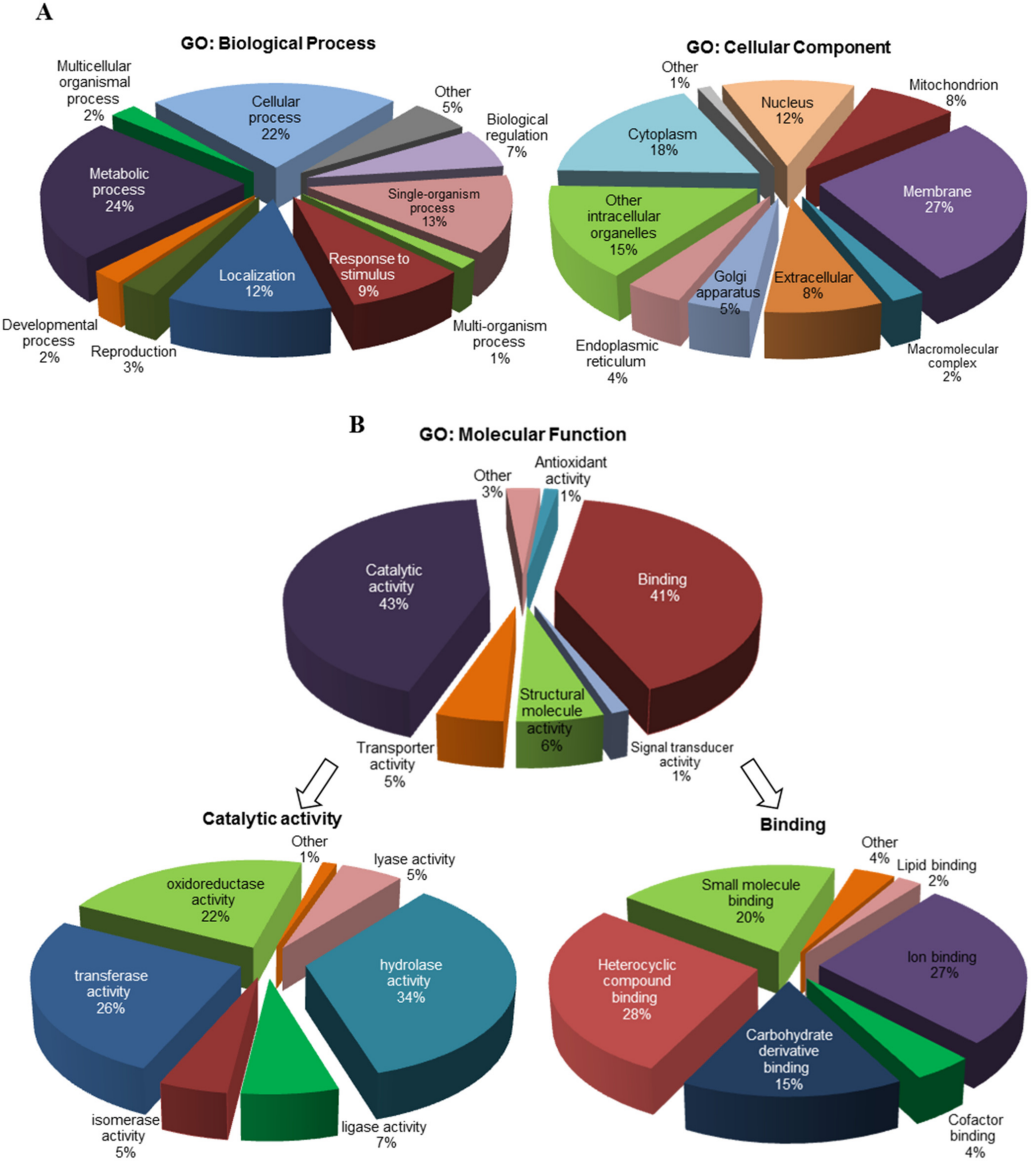
GO annotation of proteins identified in salt gland-enriched tissues of *A*. *officinalis*. A total of 1032 proteins were analysed. The proteins were classified based on GO for (A) biological process, cellular component and (B) molecular function. The major subcategories of molecular function (B) are shown in more detail on the left and right sides below the corresponding subcategories.

To better understand the functions of proteins identified in salt gland-enriched tissues, proteins that are present in at least two of the biological replicates were selected from the list of 2188 proteins for GO analysis [22]. A total of 1032 selected proteins (see S8 Table) were analysed and information were retrieved from the UniProt Knowledgebase website (http://www.uniprot.org/help/uniprotkb). The proteins were annotated based on three organizing principles of GO (Fig 3). They were characterised by their function in diverse biological processes, with 11 sub-categories identified (Fig 3A). Majority of these proteins were predicted to participate in metabolic (24%), cellular (22%) or single-organism (13%) processes or were responding to stimulus (9%), if not involved in localization (12%) (Fig 3A).

Cellular component analysis showed proteins analysed belong to 10 cellular compartments (Fig 3A). More than 70% of them were identified to be localized in membranes (27%), cytoplasm (18%), intracellular organelles (15%) or nuclei (12%) while 8% of them are extracellular proteins. For molecular function classification, 592 proteins had been assigned with 873 GO terms and seven sub-categories were identified (Fig 3B). Among them, catalytic activity (43%) and binding (41%) were the most abundant functions. Seven sub-categories were identified for proteins with catalytic activity, with majority of them (> 80%) involved in hydrolase (34%), transferase (26%) and oxidoreductase (22%) activities. For binding proteins, most were involved in heterocyclic compound (28%), ion (27%), small molecule (20%) and carbohydrate derivative (15%) binding.

**Fig 4:**
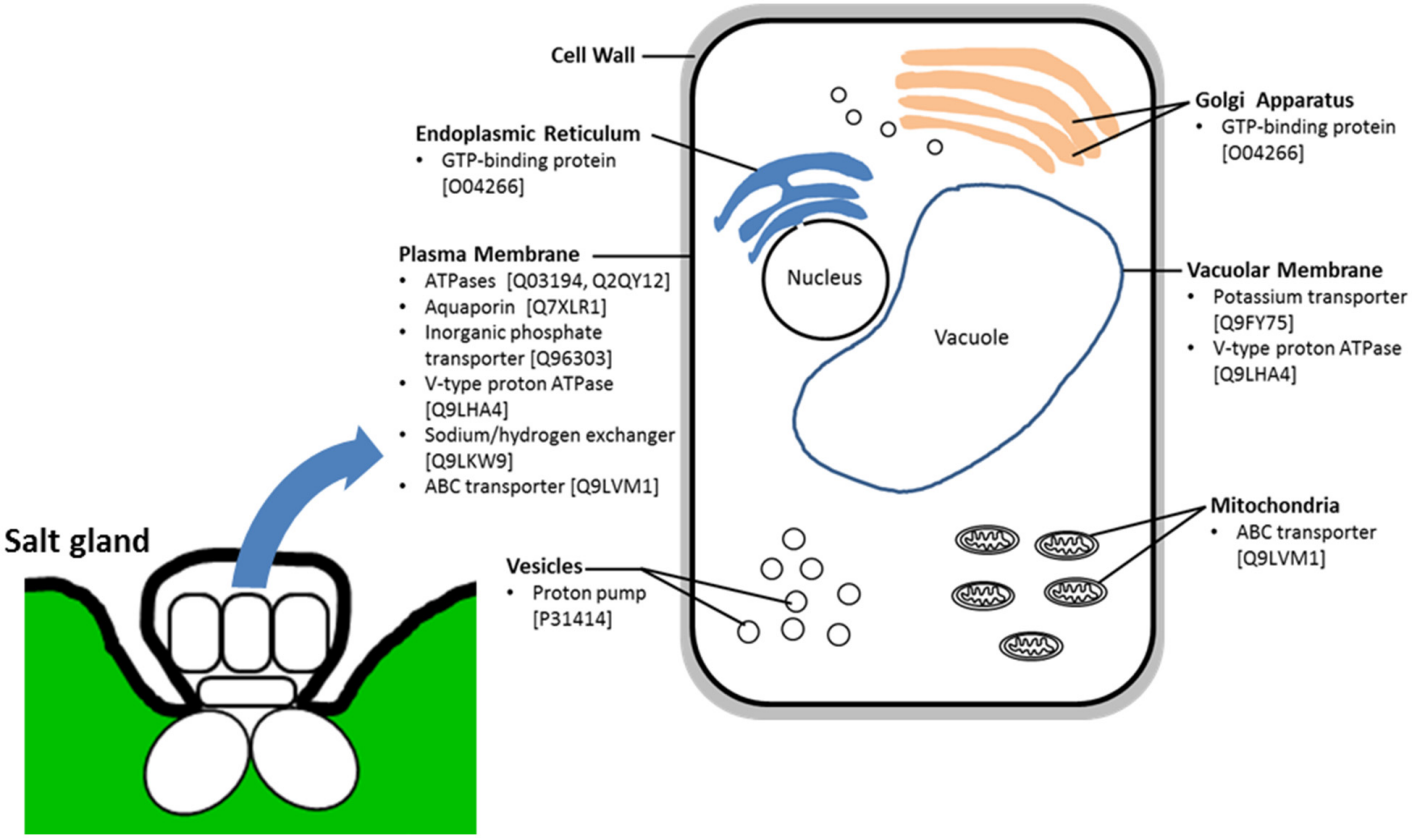
Schematic diagram of salt gland cell showing predicted cellular localization of selected list of 10 annotated proteins identified from salt gland-enriched epidermal tissues of *A*. *officinalis*. The selected proteins were classified based on GO for cellular component and Swissprot ID of the proteins are included in parentheses.

Among the list of proteins, many heat shock proteins (HSPs) or proteins related to carbohydrate and energy metabolism (e.g., ATPases, ATP synthases, aconitate hydratases, GTP-binding proteins) were identified (Table 2 and S8 Table). Proteins (Table 2). Membrane proteins such as aquaporins, transporters/exchangers, channels and pumps were also observed in the salt gland-rich tissues (Table 2). Kinases, leucine-rich repeat proteins, 14-3-3-like protein and calreticulin commonly involved in signal transduction pathways had also been identified in this study (Table 2 and S8 Table). Candidate proteins that are of interest to us pertaining to the secretory process of salt glands include ATPases (e.g., Swissprot ID: Q03194, Q2QY12), transporters (e.g., Swissprot ID: Q96303, Q9LKW9, Q9LVM1 Q9FY75), aquaporins (e.g., Swissprot ID: Q7XLR1) and GTP-binding proteins (e.g., Swissprot ID: O04266). Based on GO analysis, most of these proteins were predicted to be localized to the plasma membrane while some were expected to be found in the tonoplast, mitochondria, Golgi apparatus or endoplasmic reticulum (Fig 4).

**Table 2:** Selected list of proteins identified from salt gland-enriched epidermal tissues of *A*. *officinalis*. These proteins are identified in the epidermal tissues but not in the mesophyll tissues of *A*. *officinalis* and only those that are present in two out of the three biological replicates are selected for further anlaysis. The full list of proteins is presented in S8 Table.

				Unused ProtScore*			% Coverage^#^			Peptides (95%)^^^				
No.	gi Acession No.	Swiss-Prot ID	Contig No.	T1	T2	T3	T1	T2	T3	T1	T2	T3	Protein Description	Species
1	gi|3912949	O49996	CL15843.Contig3_All	2.27	8.00	12.21	42.90	34.30	52.70	6	5	9	14-3-3-like protein D	*Nicotiana tabacum*
2	gi|224136700	Q9LT08	CL5021.Contig4_All	7.70	3.95	4.36	32.10	33.00	22.10	6	4	3	26S proteasome non-ATPase regulatory subunit 14	*Arabidopsis thaliana*
3	gi|3914467	P93768	Unigene16875_All	2.00	2.00	0.36	30.30	30.30	30.30	1	1	1	26S proteasome non-ATPase regulatory subunit 3	*Nicotiana tabacum*
4	gi|350538091	O04059	CL2261.Contig1_All	7.52	11.08	6.18	22.30	19.20	20.80	5	6	3	3,4-dihydroxy-2-butanone kinase	*Solanum lycopersicum*
5	gi|255584390	Q9LVM1	CL12689.Contig2_All	0.91	2.66	2.00	8.90	13.80	15.40	2	1	1	ABC transporter B family member 25	*Arabidopsis thaliana*
6	gi|255552969	Q9M1H3	Unigene32800_All	4.88	5.62	2.01	18.40	17.60	18.20	4	3	1	ABC transporter F family member 4	*Arabidopsis thaliana*
7	gi|296090419	Q7PC87	CL4209.Contig1_All	4.00	2.01	21.16	44.40	46.80	53.60	8	10	13	ABC transporter G family member 34	*Arabidopsis thaliana*
8	gi|359484370	Q8RXE7	Unigene4379_All	4.83	2.00	2.18	19.20	15.10	13.60	3	1	1	ADP-ribosylation factor GTPase-activating protein AGD14	*Arabidopsis thaliana*
9	gi|255546541	Q56YU0	CL13289.Contig1_All	3.08	4.09	6.02	21.10	25.90	19.70	3	3	3	Aldehyde dehydrogenase family 2 member C4	*Arabidopsis thaliana*
10	gi|297823651	Q0PGJ6	CL5791.Contig3_All	4.87	8.40	9.74	39.20	47.70	52.30	6	7	7	Aldo-keto reductase family 4 member C9	*Arabidopsis thaliana*
11	gi|50345961	Q7XSQ9	CL16514.Contig4_All	4.00	4.01	6.03	39.50	43.00	36.00	12	12	22	Aquaporin PIP1-2	*Oryza sativa subsp*. *japonica*
12	gi|17940742	Q7XLR1	Unigene32737_All	4.00	2.09	2.00	29.60	33.70	27.60	7	5	4	Aquaporin PIP2-6	*Oryza sativa subsp*. *japonica*
13	gi|255541428		CL8427.Contig3_All	4.09	4.00	2.00	16.40	11.60	6.20	4	2	1	Arsenical pump-driving atpase	*Ricinus communis*
14	gi|2493046	Q40089	CL7437.Contig2_All	6.89	5.65	12.70	26.10	46.80	53.70	4	5	9	ATP synthase subunit delta'	*Ipomoea batatas*
15	gi|225454791		Unigene6969_All	3.38	4.00	1.54	47.10	40.20	14.90	2	2	1	ATPase family AAA domain-containing protein 3-B	*Vitis vinifera*
16	gi|224145672	P28583	CL274.Contig2_All	2.39	1.27	1.27	15.50	22.00	17.40	1	1	1	Calcium-dependent protein kinase SK5	*Glycine max*
17	gi|225466204	Q9CAL3	CL14803.Contig3_All	4.96	4.87	8.92	18.60	22.90	23.10	3	3	5	Cysteine-rich receptor-like protein kinase 2	*Arabidopsis thaliana*
18	gi|225452304	Q93ZC9	Unigene4267_All	2.33	3.39	4.71	12.10	11.20	34.00	1	2	3	Glucuronokinase 1	*Arabidopsis thaliana*
19	gi|225445585		CL12018.Contig2_All	3.12	2.33	2.04	16.20	15.80	12.00	3	1	1	GTPase-activating protein gyp7-like	*Vitis vinifera*
20	gi|351721140	O64477	Unigene30402_All	2.03	2.92	3.18	10.10	14.00	11.90	1	2	2	G-type lectin S-receptor-like serine/threonine-protein kinase At2g19130	*Arabidopsis thaliana*
21	gi|296081939	F4JMJ1	CL2876.Contig1_All	13.17	7.78	18.76	40.10	32.60	41.30	11	7	10	Heat shock 70 kDa protein 17	*Arabidopsis thaliana*
22	gi|224099789	Q9SKY8	CL2714.Contig2_All	4.14	2.01	2.44	17.60	4.20	9.10	2	1	1	Heat shock 70 kDa protein 8	*Arabidopsis thaliana*
23	gi|45331281	P09189	CL2270.Contig3_All	6.03	4.34	8.12	56.00	52.10	59.00	32	33	41	Heat shock cognate 70 kDa protein	*Petunia hybrida*
24	gi|240255879		CL2610.Contig2_All	4.06	4.00	6.00	44.80	44.80	63.20	2	2	3	Heat shock factor binding protein	*Arabidopsis thaliana*
25	gi|356540381	Q43468	CL13990.Contig2_All	2.09	6.00	2.03	53.40	52.30	40.40	4	4	3	Heat shock protein STI	*Glycine max*
26	gi|359473854		Unigene32812_All	2.14	2.09	4.00	28.70	25.30	14.30	2	1	2	hsp70 nucleotide exchange factor FES1	*Vitis vinifera*
27	gi|297793865	Q9FM19	Unigene2776_All	3.72	3.91	9.97	32.20	40.10	40.80	6	7	11	Hypersensitive-induced response protein 1	*Arabidopsis thaliana*
28	gi|255576916	O48788	Unigene6256_All	3.02	2.41	2.00	27.50	27.50	24.40	4	4	2	Inactive receptor kinase At2g26730	*Arabidopsis thaliana*
29	gi|2208908	Q96303	CL8176.Contig2_All	12.13	12.15	4.64	18.10	17.50	21.40	9	8	7	Inorganic phosphate transporter 1–4	*Arabidopsis thaliana*
30	gi|357440961	Q8GYF4	CL8176.Contig4_All	6.32	6.68	10.07	19.80	18.50	22.20	7	6	6	Inorganic phosphate transporter 1–5	*Arabidopsis thaliana*
31	gi|297740564	C0LGE0	CL3330.Contig3_All	20.94	18.55	25.32	29.50	26.70	31.40	13	12	17	LRR receptor-like serine/threonine-protein kinase At1g07650	*Arabidopsis thaliana*
32	gi|224112549	C0LGG9	Unigene4496_All	2.01	2.05	2.00	22.00	26.00	17.10	2	2	1	LRR receptor-like serine/threonine-protein kinase At1g53440	*Arabidopsis thaliana*
33	gi|359485959	C0LGH3	CL2732.Contig3_All	4.65	7.56	5.09	25.50	15.20	18.80	4	4	4	LRR receptor-like serine/threonine-protein kinase At1g56140	*Arabidopsis thaliana*
34	gi|359493576	C0LGQ5	Unigene30432_All	6.19	5.03	11.29	31.70	27.00	34.60	5	4	7	LRR receptor-like serine/threonine-protein kinase GSO1	*Arabidopsis thaliana*
35	gi|225444063		CL8211.Contig2_All	12.44	7.93	11.21	33.80	29.40	37.30	7	4	8	obg-like ATPase 1	*Vitis vinifera*
36	gi|13785471	Q9T074	CL2927.Contig4_All	22.73	13.53	24.38	36.50	26.90	40.80	17	8	16	Phosphoenolpyruvate carboxykinase [ATP]	*Arabidopsis thaliana*
37	gi|225442595	Q66GQ3	CL2133.Contig8_All	9.92	12.24	17.14	33.00	34.90	32.50	5	8	11	Protein disulfide isomerase-like 1–6	*Arabidopsis thaliana*
38	gi|225459342	Q69SA9	CL4543.Contig4_All	0.86	2.84	2.29	22.20	29.60	22.20	1	2	2	Protein disulfide isomerase-like 5–4	*Oryza sativa subsp*. *japonica*
39	gi|359494074		CL2740.Contig3_All	2.01	6.22	4.06	39.20	60.80	46.00	6	4	6	Protein grpE-like	*Vitis vinifera*
40	gi|45433315	P31569	CL4418.Contig4_All	4.00	1.76	3.06	29.60	30.10	23.60	6	5	6	Protein ycf2	*Oenothera villaricae*
41	gi|357445105	B9DFG5	Unigene196_All	2.00	6.02	7.52	22.80	20.10	41.40	6	4	4	PTI1-like tyrosine-protein kinase 3	*Arabidopsis thaliana*
42	gi|242064260	Q06572	CL6187.Contig3_All	4.00	4.01	2.00	10.10	32.40	26.30	2	3	1	Pyrophosphate-energized vacuolar membrane proton pump	*Hordeum vulgare*
43	gi|356526237	P31414	CL3527.Contig3_All	2.01	2.00	4.35	11.00	39.70	37.00	2	2	4	Pyrophosphate-energized vacuolar membrane proton pump 1	*Arabidopsis thaliana*
44	gi|359477316	Q42736	CL384.Contig14_All	4.08	10.48	2.04	18.90	21.00	21.80	6	6	6	Pyruvate, phosphate dikinase	*Flaveria pringlei*
45	gi|258678027	Q9M651	CL13915.Contig4_All	1.19	2.01	2.00	25.60	31.80	18.50	1	1	1	RAN GTPase-activating protein 2	*Arabidopsis thaliana*
46	gi|7672732	P43298	CL15187.Contig2_All	3.87	4.05	6.86	15.10	17.60	12.40	3	2	4	Receptor protein kinase TMK1	*Arabidopsis thaliana*
47	gi|224087891	Q9SCZ4	CL2320.Contig9_All	2.00	1.42	2.00	8.20	9.50	6.70	1	1	1	Receptor-like protein kinase FERONIA	*Arabidopsis thaliana*
48	gi|225427230	Q944A7	CL5141.Contig1_All	4.19	5.46	1.57	28.10	22.00	21.30	4	3	1	Serine/threonine-protein kinase At4g35230	*Arabidopsis thaliana*
49	gi|225426412	Q9FHD7	CL7376.Contig1_All	2.59	7.34	8.62	11.30	25.50	25.70	3	4	5	Serine/threonine-protein kinase At5g41260	*Arabidopsis thaliana*
50	gi|145327199	Q9CAR3	CL8777.Contig2_All	2.73	2.08	2.00	13.80	12.90	18.40	2	1	1	SNF1-related protein kinase regulatory subunit gamma-1-like	*Arabidopsis thaliana*
51	gi|194696644	Q84TI7	Unigene26174_All	2.57	2.20	2.51	24.30	38.60	37.10	1	1	1	Sodium transporter HKT1	*Arabidopsis thaliana*
52	gi|359495505	Q9LRB0	CL315.Contig4_All	2.00	0.74	2.00	11.60	17.60	6.80	1	1	1	Sphingoid long-chain bases kinase 1	*Arabidopsis thaliana*
53	gi|225443039		CL10751.Contig1_All	2.00	2.00	2.00	5.20	12.30	11.90	1	1	1	Uncharacterized membrane protein YMR155W	*Vitis vinifera*
54	gi|255542872	Q8LGG8	Unigene11881_All	0.73	1.41	2.00	12.00	16.40	22.00	1	2	2	Universal stress protein A-like protein	*Arabidopsis thaliana*
55	gi|255556366	Q8LGG8	Unigene2529_All	11.58	1.08	10.10	54.80	36.90	38.20	9	5	7	Universal stress protein A-like protein	*Arabidopsis thaliana*
56	gi|255554204	O23016	CL6704.Contig3_All	2.99	3.24	2.35	23.20	23.80	19.20	2	2	2	Voltage-gated potassium channel subunit beta	*Arabidopsis thaliana*
57	gi|357501685	Q9LHA4	CL3237.Contig3_All	9.72	1.48	12.15	18.50	22.50	21.70	6	6	6	V-type proton ATPase subunit d2	*Arabidopsis thaliana*
58	gi|225463325	Q9ZQX4	Unigene24187_All	8.32	11.16	9.34	43.90	50.00	38.50	5	7	6	V-type proton ATPase subunit F	*Arabidopsis thaliana*
59	gi|225432878	Q0WNY5	CL3494.Contig3_All	6.01	5.30	5.91	21.00	22.10	20.80	3	4	3	Wall-associated receptor kinase-like 18	*Arabidopsis thaliana*
60	gi|124221924	Q9LZM4	CL6319.Contig3_All	15.22	6.29	8.21	23.30	24.50	28.10	7	3	6	Wall-associated receptor kinase-like 20	*Arabidopsis thaliana*
61	gi|255569405	Q8RXN0	CL4681.Contig3_All	6.01	1.81		18.60	15.70		3	1		ABC transporter G family member 11	*Arabidopsis thaliana*
62	gi|171854675	P49608	CL2995.Contig26_All	2.00	2.00		29.80	14.60		1	1		Aconitate hydratase	*Cucurbita maxima*
63	gi|18076583	Q8S9L6	CL9653.Contig1_All	2.00	2.01		9.90	11.00		1	1		Cysteine-rich receptor-like protein kinase 29	*Arabidopsis thaliana*
64	gi|225445342		CL13252.Contig2_All	0.85	3.64		10.50	7.40		2	4		dnaJ homolog subfamily C member 13-like	*Vitis vinifera*
65	gi|255556438	Q9SEE5	Unigene35913_All	2.00	2.00		18.40	22.60		3	3		Galactokinase	*Arabidopsis thaliana*
66	gi|225463623		CL14543.Contig1_All	3.87	1.70		18.30	9.00		3	2		Glycerol kinase isoform 1	*Vitis vinifera*
67	gi|359497728	Q9ASS4	CL4159.Contig1_All	2.22	3.37		21.00	24.50		3	3		Inactive leucine-rich repeat receptor-like protein kinase At5g48380	*Arabidopsis thaliana*
68	gi|255552774	O04567	Unigene22230_All	1.41	0.55		8.60	19.50		1	2		Inactive receptor kinase At1g27190	*Arabidopsis thaliana*
69	gi|255586379	Q9LVM0	CL2318.Contig1_All	2.00	3.46		13.80	17.10		1	2		Inactive receptor kinase At5g58300	*Arabidopsis thaliana*
70	gi|225423806	Q9LI83	CL3370.Contig1_All	2.00	1.61		8.90	8.20		1	1		Phospholipid-transporting ATPase 10	*Arabidopsis thaliana*
71	gi|359485026	Q03194	CL16623.Contig1_All	2.16	0.83		24.60	18.20		3	2		Plasma membrane ATPase 4	*Nicotiana plumbaginifolia*
72	gi|125535713	Q2QY12	Unigene37237_All	0.79	2.00		28.40	35.80		1	1		Plasma membrane-type calcium-transporting ATPase 4	*Oryza sativa subsp*. *japonica*
73	gi|225448277	Q9FY75	CL2807.Contig2_All	2.04	2.02		4.90	4.90		1	1		Potassium transporter 7	*Arabidopsis thaliana*
74	gi|147768303	Q2MHE4	CL2457.Contig4_All	3.92	1.82		29.10	12.60		2	1		Serine/threonine-protein kinase HT1	*Arabidopsis thaliana*
75	gi|255540259	Q8LBB2	CL2181.Contig2_All	4.97	3.84		22.30	25.40		3	2		SNF1-related protein kinase regulatory subunit gamma-1	*Arabidopsis thaliana*
76	gi|350535282	Q9LKW9	Unigene18167_All	2.00	2.00		21.30	12.60		1	1		Sodium/hydrogen exchanger 7	*Arabidopsis thaliana*
77	gi|224141283	Q8LGG8	CL2994.Contig3_All	2.00	2.20		16.60	14.90		1	1		Universal stress protein A-like protein	*Arabidopsis thaliana*
78	gi|285309967	O04916	CL2995.Contig22_All	4.00		5.01	22.90		22.40	2		4	Aconitate hydratase	*Solanum tuberosum*
79	gi|224119508	Q9SYG7	CL12585.Contig1_All	2.76		2.47	11.80		15.90	3		1	Aldehyde dehydrogenase family 7 member B4	*Arabidopsis thaliana*
80	gi|225438980	O64816	CL136.Contig4_All	0.37		1.44	15.20		27.50	1		1	Casein kinase II subunit alpha	*Arabidopsis thaliana*
81	gi|224056853	P46256	Unigene21704_All	6.08		5.67	31.00		36.80	8		10	Fructose-bisphosphate aldolase	*Pisum sativum*
82	gi|6563322	O04266	Unigene25085_All	2.04		2.00	39.40		37.30	6		6	GTP-binding protein SAR1A	*Brassica campestris*
83	gi|224113157	O81832	CL3993.Contig2_All	2.01		2.00	12.80		13.90	2		2	G-type lectin S-receptor-like serine/threonine-protein kinase At4g27290	*Arabidopsis thaliana*
84	gi|225435578	Q39202	CL16541.Contig3_All	2.53		2.29	16.80		17.30	2		2	G-type lectin S-receptor-like serine/threonine-protein kinase RLK1	*Arabidopsis thaliana*
85	gi|406870037	P09189	CL2270.Contig2_All	2.78		3.55	76.20		78.10	5		8	Heat shock cognate 70 kDa protein	*Petunia hybrida*
86	gi|1708314	P51819	CL1535.Contig1_All	1.79		5.80	47.20		41.60	24		29	Heat shock protein 83	*Ipomoea nil*
87	gi|359493983	C0LGN2	Unigene56808_All	5.42		2.00	18.20		19.20	3		1	Leucine-rich repeat receptor-like serine/threonine-protein kinase At3g14840	*Arabidopsis thaliana*
88	gi|225447810	C0LGH2	CL11120.Contig8_All	2.53		2.00	24.50		25.20	1		1	LRR receptor-like serine/threonine-protein kinase At1g56130	*Arabidopsis thaliana*
89	gi|255571730	C0LGT6	CL11164.Contig3_All	0.92		6.30	26.80		23.60	1		3	LRR receptor-like serine/threonine-protein kinase EFR	*Arabidopsis thaliana*
90	gi|255546773	P98204	CL587.Contig1_All	2.00		0.48	12.80		18.50	1		1	Phospholipid-transporting ATPase 1	*Arabidopsis thaliana*
91	gi|224095202	Q9C660	Unigene8986_All	2.00		2.00	18.90		23.00	1		1	Proline-rich receptor-like protein kinase PERK10	*Arabidopsis thaliana*
92	gi|356526137	Q9LK03	CL14215.Contig2_All	2.00		2.00	26.50		20.00	1		1	Proline-rich receptor-like protein kinase PERK2	*Arabidopsis thaliana*
93	gi|302783030	Q67UF5	CL3927.Contig4_All	3.39		2.00	27.70		28.40	3		4	Protein disulfide isomerase-like 2–3	*Oryza sativa subsp*. *japonica*
94	gi|296088320	Q35638	Unigene54779_All	1.52		1.17	29.00		21.40	1		1	Rac-like GTP-binding protein RHO1	*Pisum sativum*
95	gi|359479658		CL6981.Contig1_All	2.51		4.04	23.10		20.90	2		2	Serine/threonine-protein kinase BUD32 homolog	*Vitis vinifera*
96	gi|386870491	P11796	Unigene31392_All	4.00		6.00	48.70		37.60	3		5	Superoxide dismutase [Mn]	*Nicotiana plumbaginifolia*
97	gi|359496003		CL13681.Contig2_All	1.00		1.40	7.00		24.70	1		1	Transaldolase-like	*Vitis vinifera*
98	gi|224113019	O82702	Unigene35531_All	1.55		2.09	67.50		48.10	12		9	V-type proton ATPase subunit G 1	*Nicotiana tabacum*
99	gi|44917147	O49996	Unigene34923_All		2.00	6.00		45.20	62.10		3	10	14-3-3-like protein D	*Nicotiana tabacum*
100	gi|225465653		CL3375.Contig1_All		1.80	1.22		30.60	9.60		1	1	26S proteasome non-ATPase regulatory subunit 1	*Vitis vinifera*
101	gi|225451255		Unigene49648_All		2.02	4.00		23.30	52.40		1	2	26S proteasome non-ATPase regulatory subunit 11 isoform 2	*Vitis vinifera*
102	gi|225427157	Q8LPK2	CL12866.Contig4_All		2.92	2.01		12.80	19.40		2	1	ABC transporter B family member 2	*Arabidopsis thaliana*
103	gi|296085461	Q8LPJ4	CL6385.Contig3_All		2.93	6.88		15.00	22.90		4	4	ABC transporter E family member 2	*Arabidopsis thaliana*
104	gi|2493046	Q40089	CL7437.Contig3_All		10.51	7.86		56.90	53.40		9	7	ATP synthase subunit delta'	*Ipomoea batatas*
105	gi|267631890	P28582	Unigene22237_All		2.88	8.71		28.90	28.90		4	6	Calcium-dependent protein kinase	*Daucus carota*
106	gi|11131745	P93508	Unigene55350_All		0.54	1.17		18.30	8.70		1	1	Calreticulin	*Ricinus communis*
107	gi|255566201		CL6055.Contig2_All		3.95	3.92		34.30	40.70		2	2	Co-chaperone protein HscB	*Ricinus communis*
108	gi|225462922	Q8W207	CL6827.Contig1_All		1.23	2.00		12.10	23.20		1	2	COP9 signalosome complex subunit 2	*Arabidopsis thaliana*
109	gi|225430043	Q08298	CL6583.Contig2_All		6.00	4.00		33.60	15.20		3	4	Dehydration-responsive protein RD22	*Arabidopsis thaliana*
110	gi|359477103	P22242	Unigene33129_All		8.04	5.58		19.70	37.50		4	3	Desiccation-related protein PCC13-62	*Craterostigma plantagineum*
111	gi|224120498	P21616	CL3527.Contig4_All		2.00	4.00		56.90	55.60		1	2	Pyrophosphate-energized vacuolar membrane proton pump	*Vigna radiata var*. *radiata*
112	gi|255562954	Q2MHE4	Unigene13016_All		3.03	7.85		13.30	22.20		1	4	Serine/threonine-protein kinase HT1	*Arabidopsis thaliana*
113	gi|225580057	Q9XIC7	CL10222.Contig1_All		1.66	1.00		16.50	25.90		1	2	Somatic embryogenesis receptor kinase 2	*Arabidopsis thaliana*
114	gi|75326539	Q75VR1	CL1554.Contig12_All		2.74	1.33		10.30	15.30		2	1	Two pore calcium channel protein 1A	*Nicotiana tabacum*
115	gi|148907059	Q9LHA4	CL3237.Contig1_All		7.19	1.74		20.50	25.90		6	4	V-type proton ATPase subunit d2	*Arabidopsis thaliana*

T1: first biological replicate; T2: second biological replicate; T3: third biological replicate

*: Unused ProtScore = a measurement of all the peptide evidence for a protein that is not better explained by a higher ranking protein. It is the true indicator of protein confidence.

^**#**^: % Coverage = percentage of matching amino acids from identified peptides having confidence greater than 0 divided by the total number of amino acids in the sequence.

^**^**^: Peptides (95%) = number of distinct peptides having at least 95% confidence.

## Discussion

In this study, we adopted the state-of-the-art shotgun technique to look into the proteome of salt gland-rich tissues. This approach allows the fast detection of proteins from complex mixtures and is rapidly replacing commonly gel-based methods (e.g., two-dimensional gel electrophoresis coupling with MS) [20,24,25]. Through this approach, many more proteins could be obtained (i.e, 2188 proteins identified in salt gland-rich tissues; Fig 2B and S7 Table). Among them, many of the proteins that are known to play a role in defence and stress responses in plants (e.g., stress proteins, HSPs, co-chaperones, dehydration-responsive/desiccation-related proteins, AAA-ATPase) were identified in the salt gland-rich tissues in this study (Table 2 and S8 Table). HSPs, for examples, which are one of those proteins commonly identified in this study, are members of the molecular chaperones that help to protect the plants against stress (e.g., salt stress) by refolding the proteins and maintaining their native conformation, thus preventing irreversible protein aggregation during adverse conditions [17,19,26,27]. Fructose-1,6-bisphosphate aldolase that had been identified and suggested to play a role in salt tolerance mechanisms that are common to both the glycophytes and mangrove plants [16] was also found in our salt gland-rich protein pools (Table 2). Proteins involved in signal transduction, such as 14-3-3-like protein and calreticulin that had been shown to be upregulated during salt stress in *K*. *candel* [17] and reported in the plasma membrane proteins (i.e., 14-3-3-like protein) of *A*. *officinalis* leaves [19] had also been identified herein in the salt gland-rich tissues (Table 2).

Earlier studies by Hill and Hill [28] and Faraday et al [29] looked into the ion fluxes of *Limonium* salt glands and possible involvement of ion pumps and channels during secretion had been suggested by Vassilyev and Stepanova [30]. Inhibitor studies investigating on the different types of ATPase activities or looking at various membrane proteins (channels, antiporters) during secretion had also been reported (e.g., [5,10,31–33]). Many membrane proteins (e.g., ABC transporters, sodium/potassium transporters, ATPases, aquaporins, proton pumps, sodium/hydrogen exchanger, ion channels) had been identified in the salt gland-rich protein pools (Table 2 and S8 Table) and could be involved during the desalination process. ABC transporters that are identified as one of the major transporters in our recent transcriptome study [34] and their abundance in the tonoplast and plasma membrane fractions of *A*. *officinalis* leaves [19] were also observed in the salt gland-rich protein pools in this study.

Secretion via the salt glands eliminates excess salts (predominantly Na^+^ and Cl^-^) from the plant tissues and is believed to be energy-requiring [2]. The identification of proteins associated to carbohydrate and energy metabolism (e.g., ATPases, ATP synthases, aconitate hydratases, GTP-binding proteins) in the salt gland-rich protein pools (Table 2 and S8 Table) suggest high metabolic rate within these plant tissues. Many of these proteins found in the salt gland-enriched tissues are involved in heterocyclic compound, ion and small molecule (e.g., ATP, GTP) binding (> 70%) and showed hydrolase, transferase and oxidoreductase activities (> 80%) (Figs 3 and 4, Table 2 and S8 Table) and the abundance of such proteins actually favours processes that are energy-dependent, including the desalination process in the salt glands. Determination of ATPase activities from leaves/leaf cells of salt gland-bearing species, for example, has been attempted in early studies [35,36]. Subsequent electrophysiological studies on *Avicennia* salt glands looked into possible ATPase activities [31,32]. Inhibitors of plasma membrane H^+^-ATPases (including orthovanadate) has been shown to inhibit salt secretion for both bicellular and multicellular glands [31,37]. High plasma membrane ATPase activity has been reported in the gland cells [38,39], suggesting possible role of plasma membrane P-type H^+^-ATPase in salt secretion. Recent studies on *A*. *marina* further suggest some dependence on increased ATPase and antiporter gene expression in nitric oxide-enhanced salt secretion [5].

*A*. *officinalis* under study is a salt gland-bearing tropical mangrove tree species growing towards the sea and needs to cope with ever-fluctuating salinities (0.5–35ppt) [2,40]. Taking into consideration that secretion removes not just salts, but involves an inevitable loss of water, the identification of water channels (i.e., aquaporins) at the protein level in this study further reinforce our earlier studies on the involvement of aquaporin during secretion in this species [10,34]. Salt glands of this species thus offer an excellent platform for studying dynamic responses in regulating salt and water during secretion under rapidly changing salinities. The identification of major proteins that can respond to stimulus and are involved in cellular processes enabling them to cope with dynamic salinity changes will help us understand the process better.

## Conclusion

In conclusion, we report the first proteomic analysis of salt gland-enriched tissues of a mangrove tree species. By comparing protein profiles of epidermal peels with mesophyll tissues, proteins found in salt gland-enriched tissues were identified, allowing GO analysis to be performed and a list of candidate proteins that could be involved in the desalination process identified. We believe that information obtained herein is valuable and can be used to dissect the molecular mechanisms that control the dynamics of secretion in mangrove salt glands.

The mass spectrometry proteomics data have been deposited to the ProteomeXchange Consortium (http://proteomecentral.proteomexchange.org) via the PRIDE partner repository [41] with the dataset identifier PXD000771.

## Supporting Information

S1 Table List of proteins identified in epidermal tissues of *A*. *officinalis* (first biological replicate) using AB SCIEX ProteinPilot Software 4.2, with false discovery rate (FDR) set at 1%. (XLSX)

S2 Table . List of proteins identified in epidermal tissues of *A*. *officinalis* (second biological replicate) using AB SCIEX ProteinPilot Software 4.2, with false discovery rate (FDR) set at 1%. (XLSX)

S3 Table List of proteins identified in epidermal tissues of *A*. *officinalis* (third biological replicate) using AB SCIEX ProteinPilot Software 4.2, with false discovery rate (FDR) set at 1%. (XLSX)

S4 Table List of proteins identified in mesophyll tissues of *A*. *officinalis* (first biological replicate) using AB SCIEX ProteinPilot Software 4.2, with false discovery rate (FDR) set at 1%. (XLSX)

S5 Table List of proteins identified in mesophyll tissues of *A*. *officinalis* (second biological replicate) using AB SCIEX ProteinPilot Software 4.2, with false discovery rate (FDR) set at 1%. (XLSX)

S6 Table List of proteins identified in mesophyll tissues of *A*. *officinalis* (third biological replicate) using AB SCIEX ProteinPilot Software 4.2, with false discovery rate (FDR) set at 1%. (XLSX)

S7 Table List of proteins that are identified in the epidermal tissues of *A*. *officinalis*. (XLSX)

S8 Table List of proteins from salt gland-enriched tissues selected for GO analysis. (XLSX)
